# Hybrid Optimal Design of the Eco-Hydrological Wireless Sensor Network in the Middle Reach of the Heihe River Basin, China

**DOI:** 10.3390/s141019095

**Published:** 2014-10-14

**Authors:** Jian Kang, Xin Li, Rui Jin, Yong Ge, Jinfeng Wang, Jianghao Wang

**Affiliations:** 1 Cold and Arid Regions Environmental and Engineering Research Institute, Chinese Academy of Sciences, Lanzhou 730000, China; E-Mails: kangjian@lzb.ac.cn (J.K.); lixin@lzb.ac.cn (X.L.); 2 Heihe Remote Sensing Experimental Research Station, Chinese Academy of Sciences, Lanzhou 730000, China; 3 State Key Laboratory of Resources and Environmental Information System, Institute of Geographical Science and Natural Resources Research, Chinese Academy of Sciences, Beijing 100101, China; E-Mails: gey@lreis.ac.cn (Y.G.); wangjf@lreis.ac.cn (J.W.); wangjh@lreis.ac.cn (J.W.); 4 University of Chinese Academy of Sciences, Beijing 100049, China

**Keywords:** eco-hydrological wireless sensor network, spatial sampling, hybrid optimization criterion, unconditional stochastic simulation

## Abstract

The eco-hydrological wireless sensor network (EHWSN) in the middle reaches of the Heihe River Basin in China is designed to capture the spatial and temporal variability and to estimate the ground truth for validating the remote sensing productions. However, there is no available prior information about a target variable. To meet both requirements, a hybrid model-based sampling method without any spatial autocorrelation assumptions is developed to optimize the distribution of EHWSN nodes based on geostatistics. This hybrid model incorporates two sub-criteria: one for the variogram modeling to represent the variability, another for improving the spatial prediction to evaluate remote sensing productions. The reasonability of the optimized EHWSN is validated from representativeness, the variogram modeling and the spatial accuracy through using 15 types of simulation fields generated with the unconditional geostatistical stochastic simulation. The sampling design shows good representativeness; variograms estimated by samples have less than 3% mean error relative to true variograms. Then, fields at multiple scales are predicted. As the scale increases, estimated fields have higher similarities to simulation fields at block sizes exceeding 240 m. The validations prove that this hybrid sampling method is effective for both objectives when we do not know the characteristics of an optimized variables.

## 1. Introduction

Sensor networks, one of important components of Global Earth Observation System of Systems [1], promote the advancement of Earth system science and environmental science [2]. Sensor networks, as a revolutionary technique, are widely used in several huge observation projects, such as the Critical Zone Observatories [3], the National Ecological Observatory Network [4], the Terrestrial Environmental Observatories [5] and the Long-Term Ecological Research Network [6], to understand hydrological and ecological processes.

Compared with traditional observation methods, sensor networks have two significant advantages in catchment hydrology research: the real-time monitoring of hydrological, ecological and meteorological elements and the effective capture of the spatial and temporal variability of different parameters, as well as the local spatial mean.

With the development of international water science, the NSFC (National Nature Science Foundation of China) launched the Heihe Plan entitled “Integrated research on eco-hydrological process of the Heihe River Basin (HRB)” in 2010. The Heihe Water Allied Telemetry Experimental Research (HiWATER) program is the observation platform of the Heihe Plan, and the eco-hydrological wireless sensor network (EHWSN) is one of the fundamental experiments of HiWATER [7]. The EHWSN provides indispensable observations that allow HiWATER to address problems that include heterogeneity, scaling and uncertainty. To solve these problems, EHWSN is required to capture the spatial variability and temporal dynamics of soil moisture and temperature and to provide accurate ground-truth estimates at remote sensing pixel scales [8]. Therefore, an effective EHWSN must be optimally designed.

Because of the inherent stochastic character of natural processes, researchers are frequently faced with the problem of selecting a suitable sampling location [9,10]. Geostatistics are used to capture or represent spatial variations and have been widely applied in various fields, including geoscience, water resources, environmental science and soil science [11]. A large number of spatial sampling techniques have been discussed [12] and reviewed [13]. From the perspective of geostatistics, the optimal design of spatial sampling has three objectives.

First, it aims to accurately estimate variogram parameters. Warrick and Myers [14] proposed a method (the WM criterion) in which the distribution of paired points in the lag classes corresponds to a pre-specified distribution. Müller and Zimmerman [15] and Zhu and Stein [16] focused on increasing the estimation accuracy through comparing with assumed variogram parameters.

Second, an optimal design focuses on the precision of spatial statistical inference. Optimization strategies mainly include minimizing the maximum or average kriging variance using known variogram models [17,18] or evenly distributing the samples in the study region to indirectly reduce the kriging variance. The latter strategy uses the free model without assuming variogram parameters, employing methods, such as minimizing the mean of shortest distances (MMSD) [19], maximum entropy [20], fractal dimension [21] and mean squared distance to sides, vertices and boundaries [22]. These methods, which improve spatial predictions, are only applied in ideal fields that meet the second-order stationary assumption. However, land surface variables sometimes possess stratified characteristics, especially in larger research areas, and the second-order stationary assumption does not apply in these cases. A sampling method to address the spatial stratification based on the MSN (means of surfaces with non-homogeneity) theory was proposed by Wang *et al.* [23] and Hu and Wang [24]. It estimates a variogram for each stratum and requires a larger number of samples.

Of the above methods for spatial sampling, one type for estimating variogram leads to sample clustering, which provides poor field coverage for the spatial prediction. Though the other type of method can improve spatial prediction accuracy, it is inappropriate for variogram modeling, due to the limited availability of sampling for short lag classes. To eliminate the deficiencies in both methods, the third type of hybrid criteria is proposed. It is divided into two categories. The first category is the combined use of the WM and MMSD methods [25,26]. In this category, either WM or MMSD is performed first, followed by the other, each with a specified number of samples. This is a sequential optimization process that decreases the utilization rate of the samples. It is difficult to reasonably allocate the number of samples for each sub-criterion. The second category is the simultaneous minimization of kriging variance and improving estimation of variogram parameters by assuming a known variogram [27]. However, in practice, it is difficult to know the variogram of a target variable, and the rationality of the assumption parameters cannot be evaluated.

The ENWSN is required not only to capture the spatial variability of the observed variables, but also to infer true values at the pixel scale to validate remote sensing products. Therefore, a hybrid criterion needs to be established. Because we do not know the characteristic of the specific target variable, the criterion should be based on the free model. Furthermore, to use samples more efficiently, all samples should make a contribution to each sub-criterion of the hybrid criterion. Therefore, each sub-criterion should not be performed independently, but instead, both sub-criteria should be performed together. The existing hybrid methods, however, are not suitable. To achieve both goals of the EHWSN, we develop a combination criterion without any assumptions of the spatial autocorrelation structure of surface variables. It is expressed as an integrated objective function that makes the sample distribution as uniform as possible in both geography and feature (lag distance) space.

This paper is structured as follows. Section 2 introduces the requirements of the EHWSN and the study area. Section 3 describes the optimization criterion and assessment methods. Section 4 shows the tests of the developed hybrid criterion, the final results of the spatial EHWSN distribution and validates the results with a series of evaluation indexes, and Section 5 explains the merits and remaining questions associated with this hybrid criterion.

## 2. Requirements for Optimal Sampling by EHWSN

### 2.1. Objective of EHWSN

The EHWSN in the middle reaches of HRB aims to integrate a variety of distributed ecological and hydrological observations to capture the spatial-temporal variations of key eco-hydrological variables, including soil moisture, soil temperature and land surface temperature, and to obtain the ground truth for the validation of remote sensing products over a heterogeneous land surface. To better utilize multi-source satellite/airborne remote sensing data sets in studies of eco-hydrological processes, the EHWSN employs multi-scale validation for remote sensing products using kriging technology via nested and densely-distributed WSN nodes. Optimal spatial sampling of EHWSN nodes should be performed to help achieve the above objectives.

### 2.2. Experimental Area

**Figure 1.**
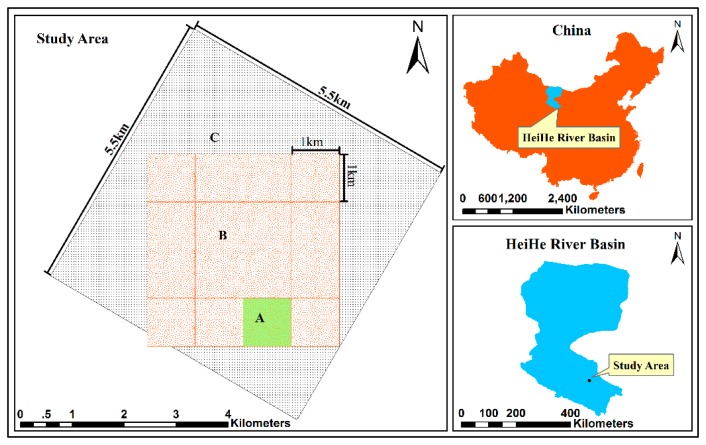
Map of the observation matrix region.

The EHWSN is installed in a 5.5 km × 5.5 km observation matrix region located in the middle reaches of HRB, which covers both the Yingke and Daman irrigation districts of Zhangye oasis, in northwest China (Figure 1). The main crop type is seed corn, covering approximately 75% of the total area. Other plants, such as wheat, vegetables and fruits, are also represented. There is a dense canal network with five types of canals that forms the area's irrigation system. This irrigation management is the main source of land surface heterogeneity.

To effectively establish a nested WSN, the observation matrix is divided into three sub-regions: (A) the intensive region, with the same area as a MODIS pixel; (B) a 4 × 4 MODIS pixels region and (C) the surrounding region.

### 2.3. Arrangement of the EHWSN Nodes

A total of 180 EHWSN nodes will be installed, including 50 WATERNET nodes, 50 SoilNET nodes and 80 BNUNET nodes. The WATERNET nodes primarily observe the soil moisture, soil temperature and soil salinity at soil layer depths of 4 cm and 10 cm. The SoilNET nodes measure soil moisture and soil temperate at soil layer depths of 4 cm, 10 cm, 20 cm and 40 cm. The BNUNET nodes also observe soil moisture at 4 cm and soil temperature at 4 cm, 10 cm and 20 cm.

**Figure 2.**
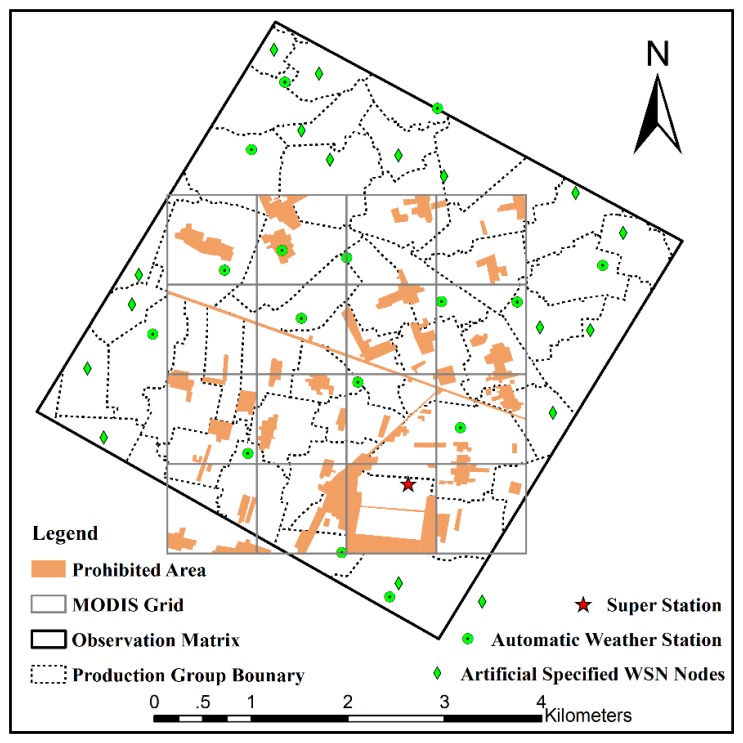
Initial eco-hydrological wireless sensor network (EHWSN) nodes in the optimization process.

There are some artificial and natural condition limitations on the spatial distribution of EHWSN nodes. In total, 17 automatic meteorological stations (AMSs) have been installed in the observation matrix to measure the evapotranspiration over the heterogeneous land surface by observing the boundary layer conditions or the flux exchange between the atmosphere and land surface. Additionally, 17 BNUNET nodes have been artificially fixed in Region C, so that there is one node per production group. These pre-specified EHWSN nodes, together with the AMSs, are considered initial points during the optimization process (Figure 2). The remaining 163 nodes are optimized in Regions A and B. Of these, 56 nodes (50 SoilNET nodes and 6 WATERNET nodes) are designed to reveal the spatial variation at a scale of hundreds of meters in Region A. The remaining 63 BNUNET nodes and 44 WATERNET nodes in Region B are used to capture the spatial variation at an approximately kilometer scale. All of the EHWSN nodes are deployed on vegetation-covered land, because the instruments cannot be installed on other land surface types, such as roads, residential buildings, wind-defended forest or irrigation channels (Figure 2).

## 3. Methodology

### 3.1. Hybrid Criterion

According to the requirements of EHWSN, we need to both effectively estimate the variogram parameters and ensure the spatial prediction accuracy when making inferences. Both requirements can be attributed to the optimal design of the sampling network; namely, how to simultaneously achieve the variogram estimation and minimizing the spatial estimation variance to the satisfied accuracy with the specified number of EHWSN nodes without assuming any variograms.

Based on the above goals, we establish an integrated hybrid model to simultaneously satisfy the two sub-criteria. This model is described by the following equation:

(1) $$\Phi_{\text{hybrid}}(S)=w_{1}\Phi_{EP}^{\text{norm}}+w_{2}\Phi_{SP}^{\text{norm}}(S)$$

where Φ*_hybrid_* is a weighted sum of two sub-criterions with weighted coefficients *w*_1_ and *w*_2_ and *S* is the optimized point set. *EP* represents a method that is effective for estimating variogram parameters, and *SP* is a method that is good for spatial prediction. Due to different dimensions, both $\Phi_{EP}^{\text{norm}}$ and $\Phi_{SP}^{\text{norm}}$ must be normalized in order to be added together.

#### 3.1.1. Sub-Criterion to Estimate the Variogram Parameters

In geostatistics, the theoretical variogram 2*γ*(*x,h*) is a function that describes the degree of spatial dependence of a random spatial field *V* [28]. The estimator 2*γ̂*(*h*) is the arithmetic mean of the squared differences between measurements *Z* at points *x* and *x* + *h*. The classical estimator of the variogram is defined as follows [29]:

(2) $$2\hat{\gamma }(h)=\frac{1}{N(h)}\overset{N(h)}{\underset{i=1}{\text{∑}}}{\left[Z(x+h)-Z(x)\right]}^{2}$$

where *N*(*h*) is the number of experimental pairs [Z(*x*), *Z*(*x* + *h*)] with distance *h* and Z(*x*) is the value at location *x*.

We choose the WM criterion [14] for the *EP* method, which relies only on the distances between the points and does not depend on assumptions of the spatial autocorrelation structure of a variable. A predefined distribution of the number of coupled pairs in all lag classes should be optimized to improve the accuracy of variogram modeling. The desired distribution can be based on expert judgment, and Russo [30] suggests that a uniform distribution can reduce the estimation uncertainty. The objective function is a simple standard deviation between the expected number of point pairs $\bar{PP}$ and the realized value in the *i*-th lag class *PP_i_*:

(3) $$\Phi_{WM}(S)=\sqrt{\frac{1}{n_{p}}\overset{n_{p}}{\underset{i=1}{\text{∑}}}{\left(PP_{i}-\bar{PP}\right)}^{2}}$$

where *S* is a set of sampling points and *n_p_* is the number of lag classes.

The function Φ*_WM_*(*S*) is normalized as a coefficient of variation (*CV*) through division by $\bar{PP}$:

(4) $$\Phi_{EP}^{\text{norm}}(S)=\frac{\Phi_{WM}(S)}{\bar{PP}}$$

where the $\bar{PP}$ value can approximated by the following:

(5) $$\bar{PP}=\frac{N_{p}}{\left(\frac{1}{2}D_{\text{max}}/LS\right)}$$

where *D_max_* is the maximum distance in the study area and *LS* the lag size. A rule of thumb is to multiply the number of lag classes by the lag size, which should equal approximately half of the maximum distance in the region. *N_p_* denotes the total number of paired points and is given by *N_p_* =*N*(*N* − 1)/2, where *N* denotes the number of samples.

#### 3.1.2. Sub-Criterion to Minimize the Estimation Variance

Normalization is difficult for the existing *SP* method based on a free model, such as MMSD, because we have little understanding of the statistical characteristics of objective function (e.g., mean, maximum and minimum). Thus, we need to develop a normalized *SP* criterion that can be compared with the WM criterion.

Yfantis *et al.* [31] confirmed that an equilateral triangle-shaped sampling network reduces estimation variance relative to a square or hexagonal network, and the best results are achieved when the regular point pattern forms equal-area Delaunay triangles. However, the measure of regularity is not sensitive to the area variance. With this in mind, a method to minimize the difference between the actual Delaunay triangle side length and the expected length (the MDS criterion) is proposed. The MDS is defined the same as in the WM criterion, *i.e.*, as a standard deviation with the following form:

(6) $$\Phi_{\text{MDS}}(S)=\sqrt{\frac{1}{n_{s}}\overset{n_{s}}{\underset{i=1}{\text{∑}}}{\left(SL_{i}-\bar{SL}\right)}^{2}}$$

where *SL_i_* is the *i*-th side length of the Delaunay triangle network generated by the point set *S*, and the Delaunay triangle diagram is calculated by Fortune [32]. $\bar{SL}$ is the desired side length. The MDS criterion is chosen for the *SP* method, and $\Phi_{SP}^{\text{norm}}$ is defined as follows:

(7) $$\Phi_{SP}^{\text{norm}}(S)=\frac{\Phi_{\text{MDS}}(S)}{\bar{SL}}$$

where Φ*_MDS_* is normalized as *CV* though division by $\bar{SL}$. Because Thiessen polygons generated by equilateral Delaunay triangles are equal-area in an infinite region, we can approximate the $\bar{SL}$ value as follows:

(8) $$\bar{SL}=\sqrt{\frac{2\sqrt{3}}{3}\times \frac{A}{N}}$$

where *A* denotes the area of study region and *N* is the total number of samples. *A*/*N* is the approximate area of the Thiessen polygons.

#### 3.1.3. Determination of Weight Coefficients

Both Φ*_WM_* and Φ*_MDS_* are converted to *CV*, and the weight coefficients *w* in Equation (1) are calculated with the following equation:

(9) $$w_{i}=\frac{CV_{i}}{\overset{n}{\underset{i=1}{\text{∑}}}CV_{i}}$$

where *w_i_* and *CV_i_* are the weight coefficient and the coefficient of variation of the *i*-th indicator, respectively, and *n* is the number of indicators. Equation (9) implies that $\overset{N}{\underset{i=1}{\text{∑}}}w_{i}=1$. The two weight values in Equation (1) are not constant; their values change with *CV_i_* during the optimization process, but their sum is equal to 1.

#### 3.1.4. Optimization Algorithm

Our goal is to develop an optimal sampling scheme with a fixed number of sampling points via minimization of the Φ*_hybrid_* value. It is necessary to find an effective way to optimize the objective function. In this paper, the simulated spatial annealing optimization algorithm (*SSA*) is employed to optimize a global sampling scheme [19,25,27,33–35]. *SSA* is a probabilistic method based on the Metropolis selection criterion [36], which can be written as follows:

(10) $$\begin{array}{cc} P_{T}(S_{i}\to S_{i+1})=1 & \text{if}\;\Phi_{\text{hybrid}}(S_{i+1})\leq \Phi_{\text{hybrid}}(S_{i})\\ P_{T}(S_{i}\to S_{i+1})=\text{exp}(\frac{\Phi_{\text{hybrid}}(S_{i})-\Phi_{\text{hybrid}}(S_{i+1})}{T}) & \text{if}\;\Phi_{\text{hybrid}}(S_{i+1})>\Phi_{\text{hybrid}}(S_{i}) \end{array}$$

where *T* represents the annealing temperature, a positive control parameter that decreases with the optimization process, and *i* is the number of iterations. The parameter *T* is calculated by the follow equation:

(11) $$T_{i+1}=\alpha T_{i}$$

where *α* is a parameter determined by users as a value slightly less than 1. In this paper, the *α* value is 0.95.

### 3.2. Generation of a Random Two-Dimensional Field for Validation

#### 3.2.1. Unconditional Geostatistical Stochastic Simulation

To evaluate the ability of this proposed hybrid criterion to describe the spatial distribution characteristics of a regional variable, a two-dimensional stationary and isotropic field with values defined on the grid (*x*, *y*) with *x* = 1, 2, ⋯, *X*; *y* = 1, 2, ⋯, *Y* is generated by stochastic simulation. The grid values are simulated using a sequential Gaussian simulation, in which ordinary kriging is used to estimate the local conditional probability distribution (LCPD) [37,38]. The simulation procedure requires the generation of spatial correlation values corresponding to a specified variogram or correlogram. Several models can be used for variogram modeling. In this study, we select the exponential model to express the spatial variation:

(12) $$\gamma (h)=c_{0}+c(1-e^{-3h/a})$$

where *c*_0_ is the nugget, *c* is the sill and *a* is the range.

Assuming that a regional variable obeys the normal distribution with mean *μ* and standard deviation *σ*, the simulated value in each grid is given by the following equation [38]:

(13) z(x,y)=xp⋅std(x,y)+mean(x,y)

where *mean*(*x*, *y*) and *std* (*x*, *y*) are the kriging-based estimated mean and standard deviation for the grid (*x*, *y*), respectively. *xp* is drawn from the standard normal distribution and computed by the following equation:

(14) xp=gauinv(p)

where *gauinv* is the numerically approximated inverse of the standard normal distribution function [39] and *p*, which represents a probability distribution function value, is a random number in the range 0–1.

The LCPD on the grid (*x*, *y*) is estimated by searching all nearby grids with known value in the dependence distance. If there are less than 10 nearby grids, a value for the grid (*x*, *y*) is randomly chosen from the normal distribution *N* (*μ*, *σ*). Each simulated datum becomes conditioning data for the next simulation step until all grids are simulated.

**Figure 3.**
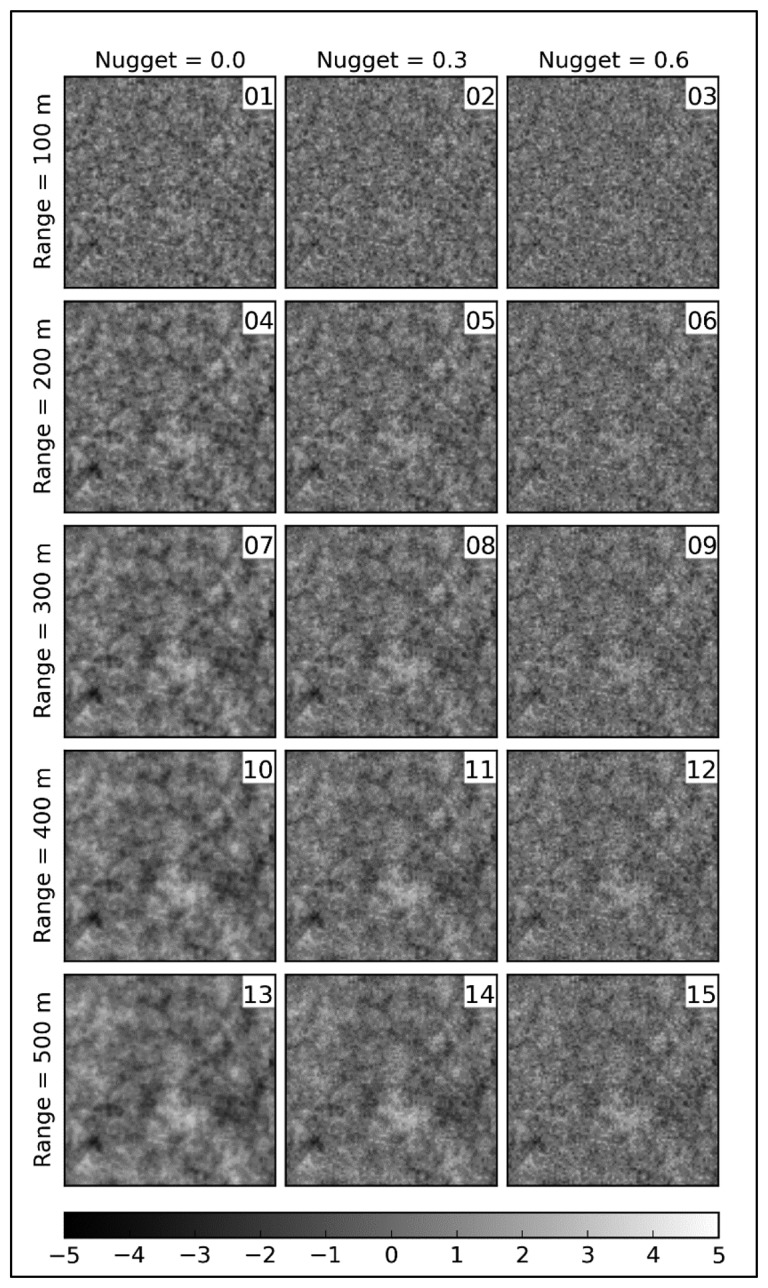
Examples of the simulation fields with 95 × 95 grids produced by the variogram with different parameter combinations.

Setting *μ* = 0 and *σ* = 1, a variogram model of *sill* = 1 is specified. The grid size is 40 m, and the number of grids is 95 × 95 for Regions A and B. In total, 50 realizations are generated for each of 15 parameter combinations of an exponential variogram model. Figure 3 shows one realization for each variogram.

#### 3.2.2. Assessment Index

To validate the representativeness of samples, the *MAE* (mean absolute error) is defined to represent the degree of bias:

(15) $$\text{MAE}=\frac{1}{n}\overset{n}{\underset{i=1}{\text{∑}}}\left|Y_{i}^{*}-Y_{i}\right|$$

where $Y_{i}^{*}$ is the prediction and *Y_i_* is the true value.

After obtaining the experimental variogram, a curve is fitted for spatial interpolation. To compare the fitted curve with the true one, the *RE* (relative error) is defined as follows:

(16) $$RE=\frac{\int_{0}^{\text{range}}\left|F(x|p_{1},p_{2},\cdots )-F^{*}(x|p_{1}^{*},p_{2}^{*},\cdots )\right|}{\int_{0}^{\text{range}}\left|F(x|p_{1},p_{2},\cdots )\right|}\times 100\%$$

where *F* and F* represent the true and fitted variograms with different parameters *p*, respectively.

For the estimated block size, the prediction accuracy is usually assessed by the block kriging variance (*BKV*) as follows:

(17) $$\text{BKV}=\sigma_{A}^{2}-\omega^{T}D-\mu$$

where *BKV* is the estimated variance for the block *A*, $\sigma_{A}^{2}$ is the area-to-area covariance over Area *A*, *μ* is the Lagrange multiplier and *D* is a vector based on the estimated point-to-area covariance. To compare with the estimated fields from the different variograms and block sizes, the mean normalized *BKV*s (*MBKV_norm_*) is calculated by the minimum-maximum normalization method:

(18) $${\text{MBKV}}_{\text{norm}}=\frac{1}{N}\overset{N}{\underset{i=1}{\text{∑}}}\frac{{\text{BKV}}_{i}-{\text{BKV}}_{\text{min}}}{{\text{BKV}}_{\text{max}}-{\text{BKV}}_{\text{min}}}$$

where *N* is the number of estimation grids. *BKV_max_* and *BKV_min_* are the maximum and minimum *BKV*s, respectively. The *BKV_i_* is the block kriging variance in the *i*-th grid.

Kriging variance only represents the estimation uncertainty, hence the similarity between the estimated field and the true field, and is defined as follows:

(19) $$F(G,S)=\frac{1}{N}\overset{N}{\underset{i=1}{\text{∑}}}(1-\frac{\left|g_{i}-s_{i}\right|}{\text{Max}(g_{i}-s_{i})})\times 100\%$$

where *G* and *S* are histograms of two images. One image is simulated by stochastic simulation, and another is estimated by kriging. The image value is divided into *N* bins. The variables *g_i_* and *s_i_* are average values in the *i*-th bin.

## 4. Results

### 4.1. Performance Test of Hybrid Criterion

**Figure 4.**
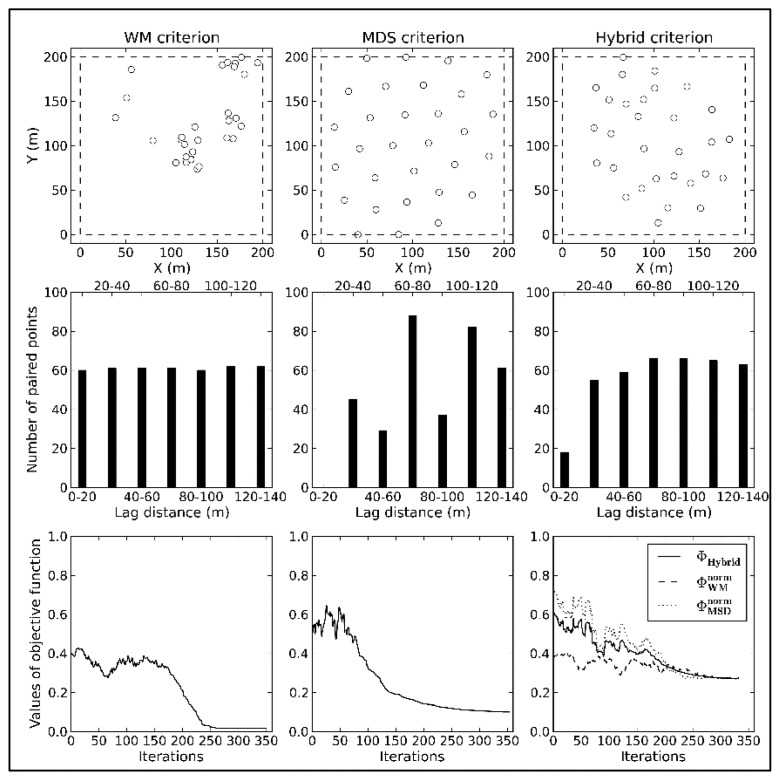
Differences in spatial distribution, number of paired points and values of objective function between three optimization criteria.

As shown in Figure 4, there is a large contradiction between the spatial distributions of the WM and MDS criteria. WM is favorable for modeling spatial variation, but causes excessive aggregation, which negatively affects spatial estimation. MDS insufficiently captures the spatial variation due to the lack of information at short lag classes. Additionally, points near the boundaries do not have enough neighbors, leading to biases at the corners in MDS. This problem can be solved though initializing a few points in the corners.

The results of the hybrid optimal criterion are more or less similar to designs that are optimized by either WM or MDS. Though the hybrid model does not perfectly inherit all advantages of the sub-criteria, their defects are remedied. Compared to WM, the spatial distribution of samples generated by the hybrid criterion is superior for spatial statistical inferences, and compared to MDS, the distribution of samples in lag space is more complete.

Because the initial values of both sub-criteria are different, the spatial distribution of samples will tend toward the criterion with the smaller initial value if equal weights are assigned to each sub-criterion. In this test, the final results will tend toward the WM criterion. Hence, variable weight coefficients are reasonable during the entire optimization process.

### 4.2. EHWSN Optimization15

**Figure 5.**
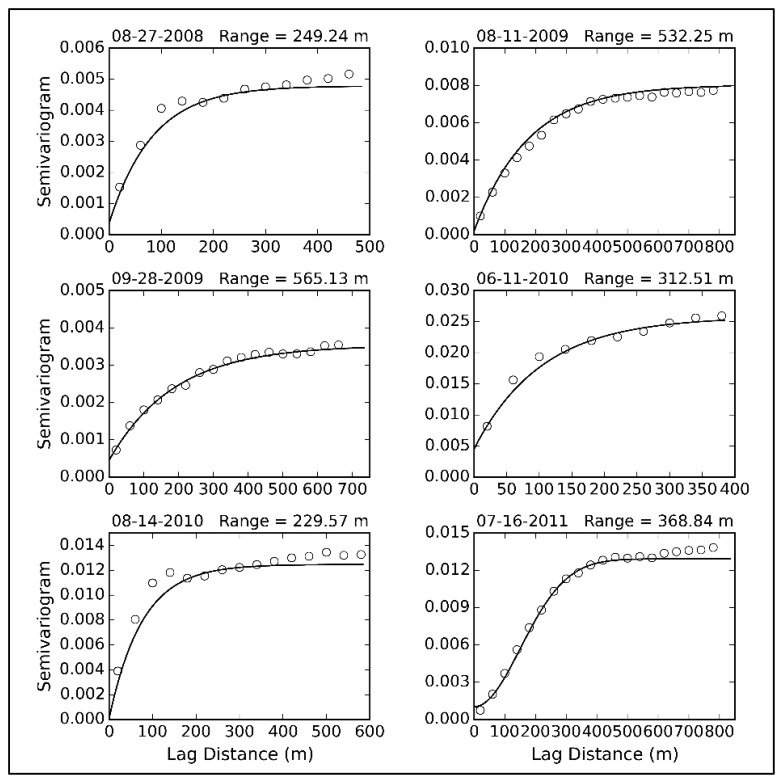
Experimental variogram (circles) and fitted curve (black line) of the Temperature-Vegetation Dryness Index (TVDI).

**Figure 6.**
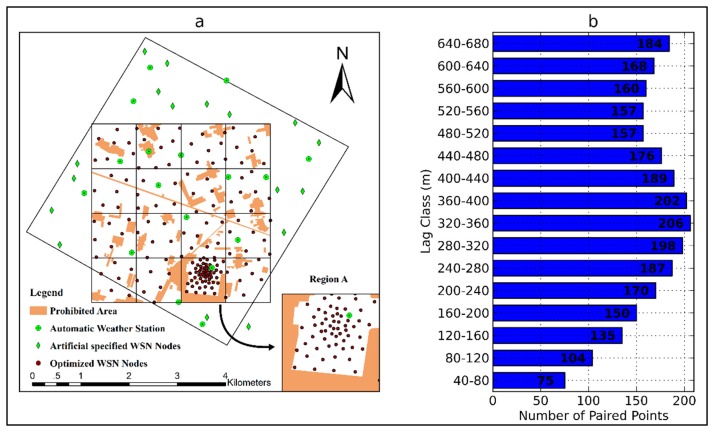
Optimized distribution of EHWSN nodes in the observation matrix (**a**) and the number of paired points in each lag class (**b**).

We have proposed a hybrid model-based optimization method with two sub-criteria for parameter estimation and spatial statistical inference. This method is applied to the EHWSN sampling design in the middle reaches of HRB. The relevant parameters are as follows: $\bar{PP}$ in Equation (5) is equal to 200 and $\bar{SL}$ in Equation (8) is equal to 130 m in Region A and 380 m in Region B. The size of each field block is approximately 40 m × 40 m. To avoid having more than one node in a field block (only the variability between fields is considered), a pair of points separated by less than 40 m is forbidden. The lag separation distance should coincide with the field spacing; thus, the lag size is set equal to 40 m. Lag classes less than the dependence distance should be given priority. However, it is difficult to select a suitable distance. Therefore, we attempt to obtain the dependence distance using the TVDI (Temperature-Vegetation Dryness Index) derived from a Thematic Mapper (TM) image. This image substitutes for soil moisture [40], because there is no prior high-resolution information of the soil moisture distribution. The TVDI can approximately represent the spatial distribution of soil moisture. Ultimately, the dependence distance is set equal to 680 m (Figure 5). The final result is shown in Figure 6. Optimization decreased the objective function Φ*_hybrid_* from 0.84 to 0.28. The final values of $\Phi_{EP}^{\text{norm}}$ and $\Phi_{SP}^{\text{norm}}$ are 0.24 and 0.31, with weight coefficients of 0.44 and 0.56, respectively.

### 4.3. Validation

The optimization results of EHWSN need to be verified using simulated fields from several perspectives, including the representativeness, the accuracies of parameter estimation and spatial prediction.

#### 4.3.1. Representativeness

**Figure 7.**
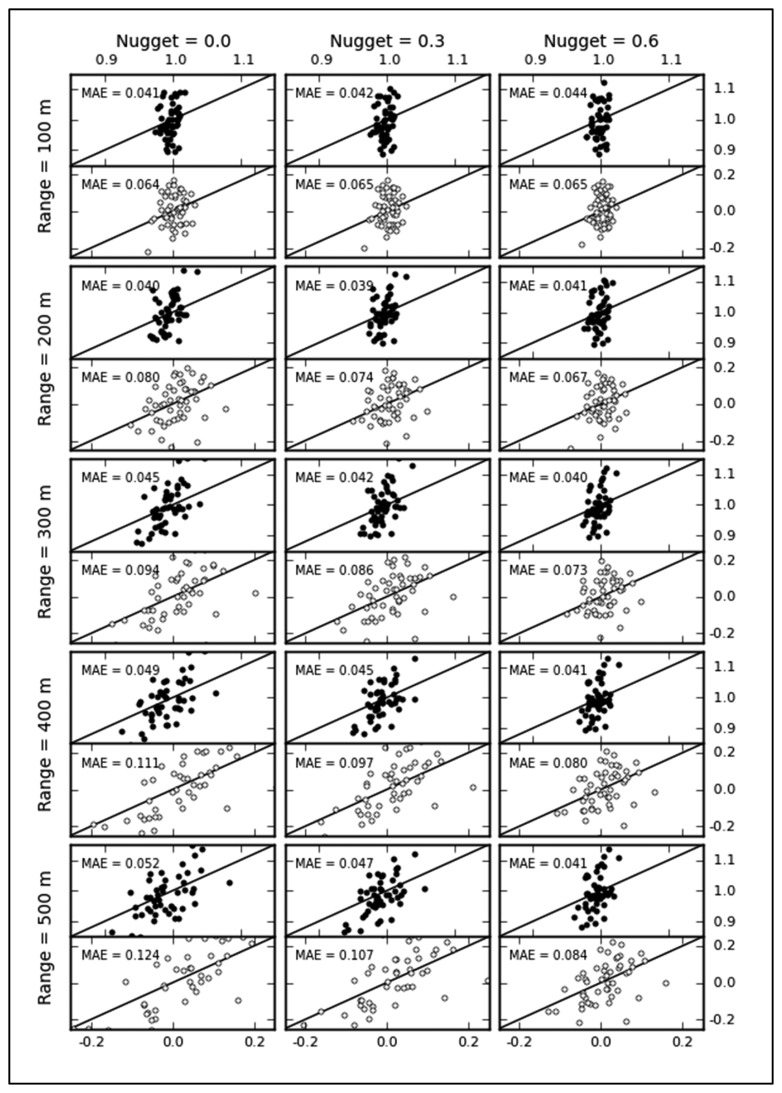
Scatter diagrams of the means (circles) and standard deviations (black dots). The *Y*- and *X*-axes represent the samples and the population, respectively.

The inferred values of target variables from spatial predictions at unsampled locations are based on the hypothesis that samples are representative. If data are sampled in an unrepresentative manner, the biased data cannot represent the overall properties in the area of interest, and the spatial predictions based on geostatistical techniques might be worse than those based on simple methods, such as inverse distance interpolation, surface interpolation and splines. To verify the representativeness, we compare the means and standard deviations (SD) between the samples and the populations from 50 stochastic realizations of each of 15 variogram parameter combinations. *MAE* values for mean and SD closing to zero indicate spatially representative samples. As shown in Figure 7, the scatterplot represents the degree of biases for 15 types of simulated fields. The final optimization results in the middle reaches of HRB exhibits good representativeness, and the maximum *MAE* is approximately 0.12. The representativeness of samples gradually increases with increasing the nugget and decreasing range. Intuitively, samples from fields with smaller nuggets and larger ranges should be more representative, but the results in Figure 7 are the opposite. This is because there are intensive observation nodes in Region A. Table 1 lists the *MAE*s of samples in Regions A and B. Large differences are observed between both regions. Regardless of the variability, samples in Region B with relatively uniform spatial distributions have low *MAE* values for different simulated fields. The *MAE* index is sensitive to the variability in regional variables when the spatial distribution of samples is excessively concentrated. Although cluster observations may lead to a greater degree of deviation from the population, the local cluster points is necessary for estimating variogram parameters.

#### 4.3.2. Parameter Estimation

To capture the spatial variability, a specified number of paired points for 16 lag classes (Figure 6) are optimized to estimate the variogram. According to previous experience, at least 50 paired points are required [41]. Pairs of points in lag classes of 60 m, 100 m, 140 m, 180 m and 220 m are generated in the intensive Region A.

**Figure 8.**
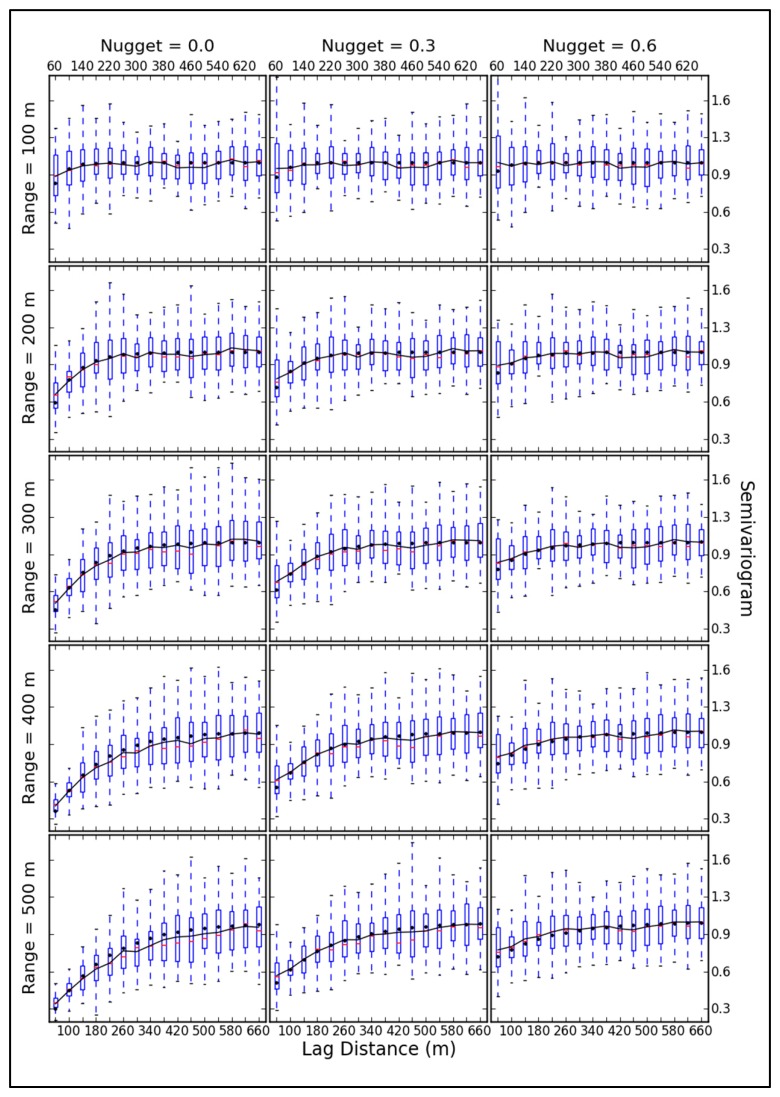
Experimental (black line) and theoretical (black dots) variograms. The boxplot represents the dispersion for the experimental variogram. The boxes with lines denote the median (thick line), lower quartile and upper quartile values (dotted lines).

The dispersion and mean of the experimental variogram at different scales is described in Figure 8. With variations in the nugget and range, differences are generated in the dispersion at different scales. Small scales (60 m, 100 m and 140 m) especially exhibit large changes in dispersion. When the range increases, the dispersion of small scales decreases, because of the increased structure property, while at larger scales (>140 m), the dispersion increases slightly. At all estimated scales, the dispersion changes due to nugget variations are the opposite of the changes due to variations in range.

The trend of the average line of the experimental variogram is close to the theoretical function, and it excellently expresses the spatial variability. When the structural characteristic is dominant (the proportion of the partial sill to sill is larger than that of the nugget to sill), the biases between theoretical and experimental variograms is low at small scales and high at large scales. However, with the growth of randomness, the biases at estimated scales are opposite.

To test the accuracy of estimated variogram parameters, the *RE* between the true variogram and the fitted curve is calculated (Table 2). For a dependence distance of 100 m, the estimated parameters are unavailable, because only one experimental variogram value in the distance of less than 100 m is used to fit the curve. With that exception, the relative error increases with increases in range when there is no non-spatial variance (Nugget = 0). When the major component of variance is non-spatial (Nugget = 0.6), the relative error of simulated fields decreases with increasing range. The maximum relative error is less than 6%, and the mean relative error is 3%.

#### 4.3.3. Spatial Prediction

**Figure 9.**
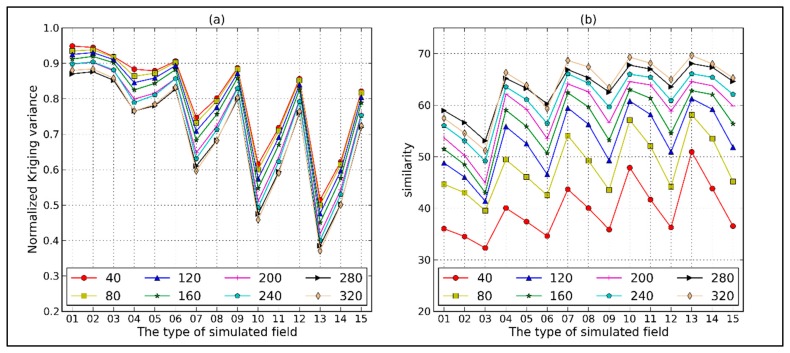
Normalized kriging variance (**a**) and similarities (**b**) between the simulated and estimated fields with different block sizes.

After estimating the variogram, the interpolation accuracy also needs to be evaluated. Generally, the interpolation accuracy is evaluated by the kriging variance, which expresses the estimated uncertainty. We apply block kriging to obtain the estimated fields with eight grid sizes ranging from 40 m to 320 m. If the variogram parameters and estimated block sizes have been determined, the kriging variance is only related to the spatial distribution of samples and is independent of the sample values. As shown in Figure 9a, for each type of estimation field, the mean normalized kriging variances (*MBKV_norm_*) in Equation (18) become small with increasing estimation scale. This is the spatial variance within a larger block that is cancelled out, leaving less uncertainty. For each estimation scale, the *MBKV_norm_* becomes large with decreasing dependence distance or increasing nugget value, which leads to the growth of uncertainty.

Kriging variance, however, cannot express the biases between true and estimated values. Therefore, we use another index to evaluate the estimation accuracy. The similarity between the simulated field and the estimated field with same block sizes is calculated by Equation (19). The similarity changes with estimation scales and parameters of variograms is opposite of the *MBKV_norm_* changes. As Figure 9b shows, the estimated field with the lowest randomness and maximum estimation scale (320 m) shows a maximum similarity of about 70% to the true field. In contrast, the estimated field with the strongest randomness and minimum estimation scale (40 m) shows a minimum similarity of about 32%.

## 5. Conclusions and Discussions

A hybrid sampling method is proposed to optimize samples for both spatial estimation and spatial interpolation when there is lack of prior information on the target variable. This hybrid method is used to optimally design the EHWSN in the HRB, and its effectiveness has been verified in terms of representativeness, parameter estimation and prediction accuracy, using various simulation fields.

The samples collected by the hybrid sampling method show the excellent representativeness. The relatively even spatial sampling in Region B enhances the representativeness of samples for different types of simulation fields. Though the nested samples in Region A introduce a slight sampling bias, it can improve the estimation accuracy of variogram parameters at small scales.

For variogram modeling, the stronger a regional variable shows spatial randomness, the more paired points are needed to capture the variability at small scales. When the structural features of a regional variable are obvious, more paired points are needed at larger scales. In this research, reliable prior information about the target variable is unavailable; therefore, using the equal treatment of paired points in each lag to estimate the variogram parameters is reasonable.

One of our objectives is to estimate ground truth at different remote sensing pixel scales. Both accurate parameter estimation and high sample representativeness are helpful for achieving this goal. The sampling design for estimation at block sizes exceeding 240 m has higher similarities. The differences of fluctuations between similarity curve lines with different block sizes indicates the influences of variogram parameters variation on the estimation accuracy. With the growth of the estimation scale, the amplitudes of fluctuations become small, which means that variogram parameters have less impact on the estimation accuracy. This information is meaningful, and if we estimate a quite large block, it is not necessary to be overly concerned with the estimation of variogram parameters. Instead, the sampling design may make more contributions to enhance the representativeness of samples.

For meeting multi-scale estimation requirements, the nested structure is designed. However, the cluster points may lead to some problems, such as decreasing the representativeness of the samples, enhancing the bias in the estimated variability at small scales and bringing a negative effect on the spatial prediction. Ideally, the multi-cluster sampling, that points with a uniform spatial distribution are combined with multi-cluster points evenly distributed across the study area, can effectively eliminate these problems. However, such a sampling design needs to encompass a large number of samples. Due to budget limitations, only one point cluster is produced in our experiments. The validations prove that the nested sampling design is effective for both variogram modeling and spatial prediction based on limited samples.

In addition, unbiased sampling is important in the optimal design. In this work, the hybrid criterion considers both parameter estimation and spatial statistical inference. However, there is no quantitative expression to represent in the objective function. Therefore, future research should investigate how to quantify representation during the optimization process.

## Figures and Tables

**Table 1.** Biases of the means and standard deviations between the samples and the population.

**Nugget**	**0.0**				**0.3**				**0.6**			
			
**Range**	**Region A**		**Region B**		**Region A**		**Region B**		**Region A**		**Region B**	
					
	**Mean**	**SD**	**Mean**	**SD**	**Mean**	**SD**	**Mean**	**SD**	**Mean**	**SD**	**Mean**	**SD**
100 m	0.138	0.059	0.071	0.055	0.130	0.063	0.074	0.055	0.121	0.068	0.076	0.055
200 m	0.212	0.079	0.063	0.054	0.185	0.066	0.067	0.053	0.151	0.062	0.072	0.053
300 m	0.271	0.105	0.060	0.053	0.231	0.083	0.065	0.051	0.179	0.065	0.071	0.051
400 m	0.325	0.133	0.058	0.052	0.272	0.101	0.064	0.050	0.201	0.072	0.070	0.050
500 m	0.368	0.160	0.056	0.050	0.312	0.116	0.063	0.049	0.218	0.076	0.069	0.050

**Table 2.** Accuracy of parameter estimations.

**Nugget**	**0.0**				**0.3**				**0.6**			
			
**Range**	**Nugget** *	**Sill** *	**Range** *	***RE***	**Nugget** *	**Sill** *	**Range** *	***RE***	**Nugget** *	**Sill** *	**Range** *	***RE***
100 m	0.63	0.99	82.59	3.98%	0.88	0.99	181.61	3.59%	0.99	0.99	99.96	2.76%
200 m	0.31	1.00	262.33	2.54%	0.55	1.00	258.71	2.21%	0.75	0.99	220.66	1.86%
300 m	0.21	1.01	395.43	2.87%	0.46	1.00	368.75	1.95%	0.69	0.99	300.00	1.80%
400 m	0.16	1.00	516.82	4.58%	0.42	0.99	473.47	2.34%	0.69	0.99	422.18	1.69%
500 m	0.13	1.00	662.94	5.69%	0.40	0.99	589.32	2.71%	0.67	0.99	483.18	1.63%

Note:

* means the estimators of variogram parameters.
